# Supplementary material for: Visualization and analysis of RNA-Seq assembly graphs
Source: Nucleic Acids Res. 2019 Jul 15;47(14):7262–75. doi: 10.1093/nar/gkz599 (PMC6698738; doi:10.1093/nar/gkz599)
Supplement: gkz599_Supplemental_Files [file gkz599_supplemental_files.zip › Supp. file 1 - protocol.docx]

## Protocols for generating a networks from RNA-seq data

This protocol provides details on how to generate a RNA assembly network file through a number of different options:

1. **Website –** allows a user to generate networks from predefined RNA-seq datasets; NHDF fibroblast samples taken 0, 12, 18 and 24 h after serum refeeding; or derived from 27 different human tissue samples (ArrayExpress Ac.No.: E-MTAB-1733).
2. **UNIX –** allows a user to run the pipeline on their own dataset on local servers using the code accessible from our GitHub repository.
3. **Amazon cloud –** for a user who has an Amazon account can simply run the pipeline from Amazon Machine Images (AMI) of Amazon Web Services (AWS).

The final section **(D)** deals with the visualisation and analysis of DNA assembly network files.

**
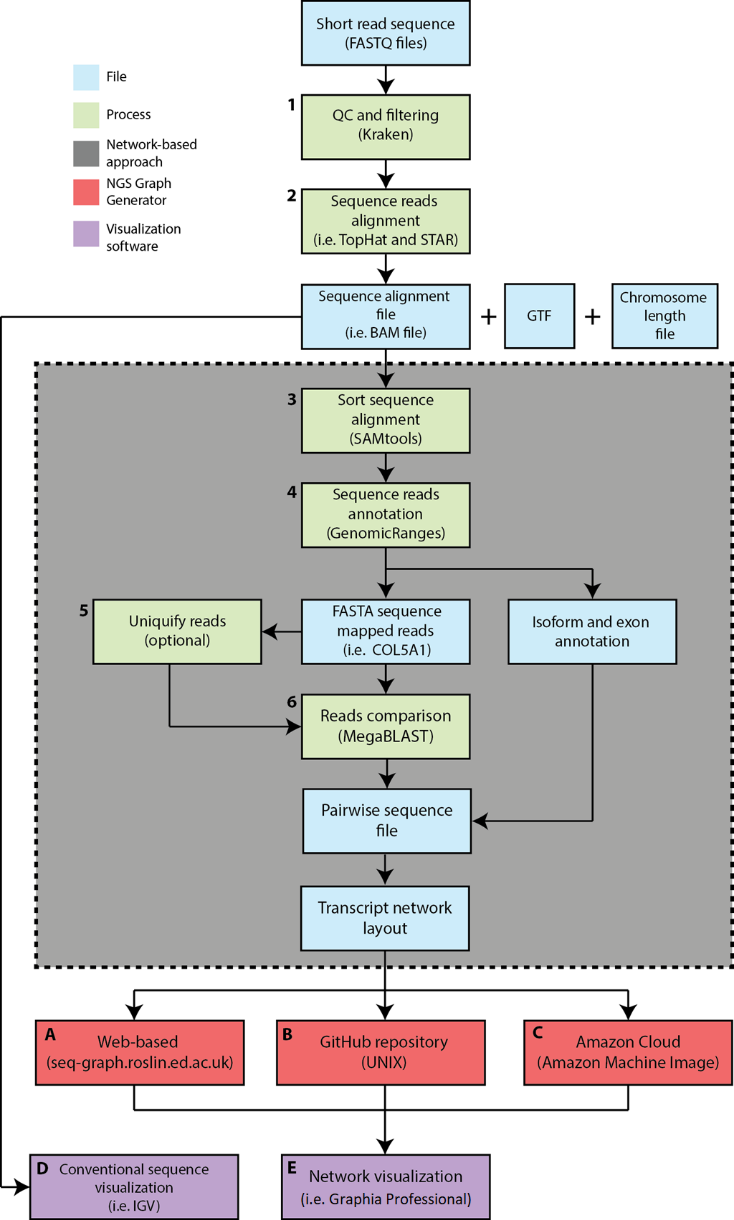
**

**Figure 1: Overview of the pipeline for network-based visualisation of RNA-seq data**. Schematic of the pipeline used for building RNA assembly networks from FASTQ files through to network visualisation within Graphia Professional. **(1)** Short read sequence data, i.e. FASTQ files from the NGS sequencer are used as the input for this pipeline, followed by quality control for which the Kraken pipeline is recommended. **(2)** Reads are aligned to a reference genome using TopHat or STAR. **(3)** Output from the aligners is a BAM file which is used as an input for the NGS Graph Generator pipeline together with a GTF and chromosome length file. The BAM file will subsequently be sorted based on the genome coordinates using SAMtools. **(4)** This is followed by read annotation using *GenomicRanges*. The outputs of this step are FASTA sequence of mapped reads and the nodeclass file of exon and isoform annotation for each sequence read. **(5)** Depending on the depth of sequencing (gene expression level), an option to remove redundant reads can be used. **(6)** Read-to-read comparison of all short sequence reads of selected gene/locus using MegaBLAST producing a read-to-read network file. This together with the output of genomic ranges is mergef to produce a layout file. This NGS Graph Generator (grey box) is incorporated into three different platforms, **(A)** web-based, **(B)** GitHub (UNIX) and **(C)** Amazon Cloud. results can be compared with the network-based visualization approach using Graphia Professional.

### Website

1. Go to the website: <https://www.seq-graph.roslin.ed.ac.uk>
2. There are a number of precomputed example files available in the ‘Gallery’ section, accessable when you click on an image of a gene network. Notes on how to visualise the network are given in section D of this document.
3. To generate a new gene assembly network, navigate to the website of NGS Graph Generator. Click on ‘Generate A Graph’ on the top menu of the home page.
4. Select an RNA-Seq dataset and type in your gene of interest, e.g. TPM1. You may also choose to change the default Mega BLAST settings for the percentage sequence length and similarity, and reference files. Finally, you may chose to discard identical reads, thereby reducing the resultant network’s size, assuming there was read redundancy in the first place. When finished enter you email and click ‘Schedule job’.
5. Validate your email if you are using the NGS Graph Generator website for the first time. Once your email has been validated, you can schedule further jobs.
6. Once a job has finished, an email will be sent to you with a link. Download the layout file by clicking the ‘green’ box of the gene name (i.e. TPM1-s98-l31.layout or job-101.gz). You are now ready visualise the results – see section D.

**Please note:** Run time of this pipeline on the sample data provided will depend on the number of reads mapping to the selected gene and use of server at the time of scheduling the job.


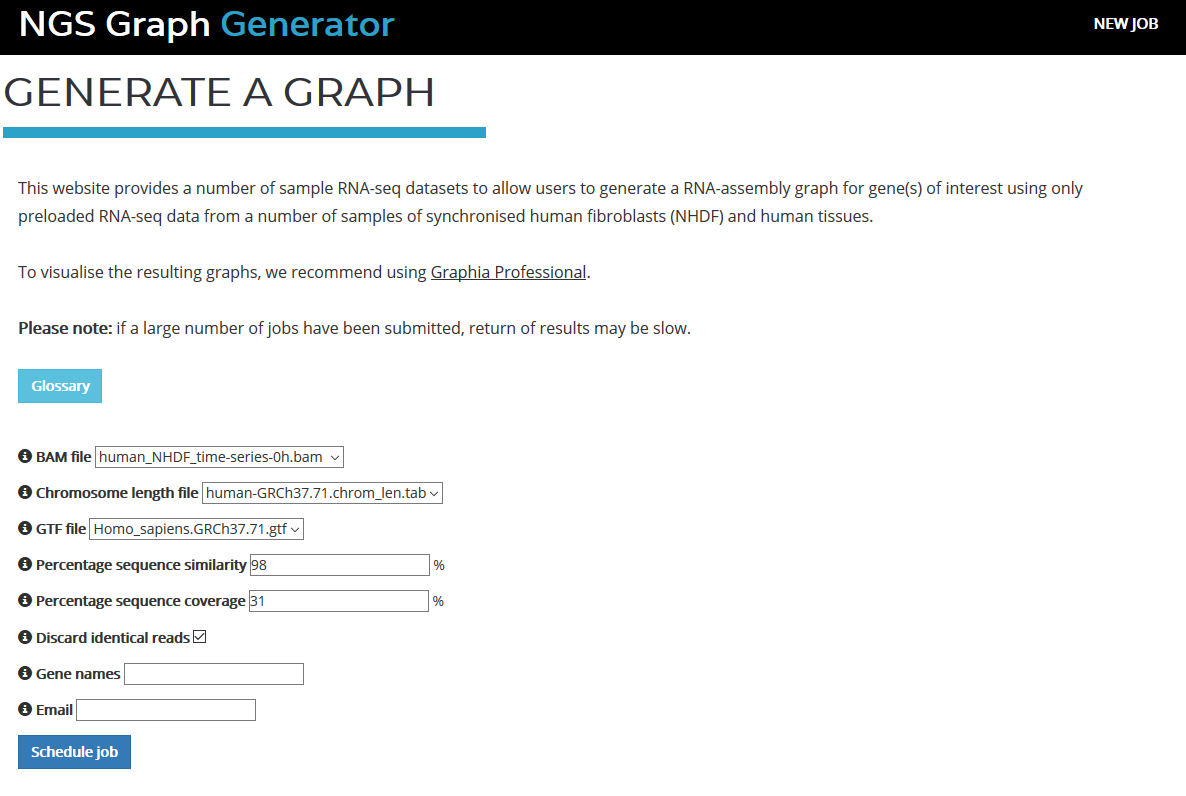


**Generate a graph page:** <http://seq-graph.roslin.ed.ac.uk/generate.php>. Once you open ‘Generate A Graph’ from the home page, this page will appear. Select one of the RNA-seq datasets provided; human fibroblasts 0, 12, 18 and 24 hours after serum re-feeding or data from one of 27 human tissues. As the provided datasets are all from human, the ‘Chromosome length file’ and ‘GTF file’ options should be left as they are. The parameter of read-to-read comparison ‘percentage length similarity’ and ‘percentage sequence coverage’ has been set to a default value which is 98% and 31%, respectively but can be altered. The removal of redundant reads is also a default. Type in the symbols for the gene or genes of interest in the field of ‘Gene name’ (e.g. COL5A1 or COL5A1, BUB3, TP53). Lastly, provide your email.

### Running the pipeline on UNIX

1. Download and install the packages:

- Python version ≥ 2.7.3 (<https://www.python.org/>)
- SAMtools version ≥ 0.1.18 (<http://samtools.sourceforge.net/>)
- MegaBLAST version ≥ 2.2.19 (<http://blast.ncbi.nlm.nih.gov/>)
- R version ≥ 2.15.2 (<http://www.r-project.org/>)

*Note:* Ensure the R installation also includes these R packages

- - *GenomicRanges*
  - *Biostrings*
  - *Getopt*

To check the R packages are installed, in the R environment type:

lib(GenomicRanges).

If it is not installed, please install it by typing a command:

$source("https://bioconductor.org/biocLite.R")

$biocLite(“GenomicRanges”)

(try http:// if https:// URLs are not supported)

1. Install RNA-seq pipeline package. Get the pipeline package from the Github repository <https://github.com/systems-immunology-roslin-institute/ngs-graph-generator>. The package can be accessed in two ways; clone with HTTPS or download ZIP. The first choice can be performed by clicking the ‘Clone or download’; copy the link provided and clone the package into the server. Should you have trouble refer to GitHub Help at <https://help.github.com/articles/which-remote-url-should-i-use/>. The second option can be achieved by clicking the ‘Clone or download’, click ‘Download ZIP’ button and save it to your server. Unzip the file ‘ngs-graph-generator-master’ as shown below:

$ gzip –d ngs-graph-generator-master.zip

Change directory into ‘ngs-graph-generator’ folder

$ cd ngs-graph-generator-master/ngs-graph-generator

For the purpose of testing the pipeline, a small dataset is provided for you to run. Download the test (*LRR1* gene) BAM file here:

$ <http://seq-graph.roslin.ed.ac.uk/testdata.bam>

1. Input files. Once the necessary software and tools are installed, you must prepare the files needed prior to running the NGS Graph Generator pipline. The input details are as follows: a) mapping file from the alignment output (BAM file) generated by a program such as TopHat or STAR; b) GTF/GFF file (the same GTF/GFF file used when running the alignment); c) Chromosome length file. The file is a lists the size of each chromosome in base pairs (bp).

There are two possibilities in creating a network layout; FASTQ files or mapping files. If you have FASTQ files which are short read sequence files from an NGS sequencer, you should run the quality control check by using Kraken pipeline. It is a fast and efficient tool to process the FASTQ files. Subsequently, the filtered FASTQ is aligned to reference genome using TopHat or STAR alignment. GTF/GFF file is needed as input for this pipeline. The file should be the same file used in performing the alignment. The last file needed as an input is a chromosome length file. Different versions of Ensembl GTF report different sizes for chromosomes.

1. Creating a network layout. The time to create a network layout largely depends on the length and expression level of the selected gene, i.e. how many reads map to it.

To run the pipeline, type the command-line:

$ create-biolayout-file.sh –b <bam file> -t <The chromosome length file> -g <The GTF file> -o <The directory in which to place the output> -d <"GENE" A list of genes to examine> -p <The percentage similarity value> -l <The percentage coverage value> -u <discard redundant reads>

Where:

–b is a BAM file after the mapping process

–t is a chromosome length file

–g is a GTF/GFF file for annotation of the nodes

–o is the desired output directory of this process

–d is a gene or set of genes for the analysis

–p is a percentage sequence similarity (overlap) between two reads (default = 98)

–l is a percentage coverage value (default = 31)

–u is an optional instruction to remove redundant reads

1. **Run on test data**

Data has been generated using Illumina HiSeq 200 from human fibroblasts 24 hours after serum re-feeding. Data was aligned using the alignment software, TopHat. The gene used in this protocol is *LRR1.* We recommend that you use unsorted BAM files. The input file below is a small BAM file for the purpose of this protocol. To ensure the file is in the directory, type a command as shown below:

$ ls testdata.bam

Ensure all the necessary files are in the current directory. Run the pipeline by typing the command line as shown below:

$ ./create-biolayout-file.sh –b testdata.bam –t human-GRCh37.71.chrom_len.tab –g testdata.gtf –o output –d LRR1 –p 98 –l 31

If the selected gene is highly expressed, an option of –u is recommended to remove the read redundancy.

Once the job is finished, change to output directory by typing:

$ cd output

The output of this pipeline will be in the layout file format:

$ LRR1-s98-l31.layout

The output name means:

- - LRR1 – the selected gene
  - s98 – percentage length similarity
  - l31 – percentage length coverage
  - .layout – a layout format which can be open using Graphia Professional (formally known as Biolayout Express^3D^)

This file is ready to be visualized using network visualization tool. Refer to **Section D** for visualising the output from this pipeline.

### Amazon cloud

An Amazon Machine Image (AMI) provides the information required to launch an instance – a virtual server in the cloud. You specify an AMI when you launch an instance, and you can launch as many instances from the AMI as you need. To run NGS Graph Generator on the Amazon Web Services (AWS) Marketplace, you need to create an AWS account. NGS Graph Generator is eligible for the [AWS Free Tier](http://aws.amazon.com/free/), which is available to new users of Amazon Web Services Marketplace.

|  |  |
| --- | --- |

1. Sign-in to [AWS Marketplace](https://aws.amazon.com/marketplace/)**.** You need to have an Amazon Cloud account in order to create a network layout from the server. If you are new to AWS, see [Getting Started with AWS](https://aws.amazon.com/getting-started/?nc2=h_l2_cc) for information about how to create an account. Create your AWS account here <https://aws.amazon.com/free/>
2. Create virtual server. Next, you have to create a virtual server using elastic cloud 2 (EC2). Under ‘Compute’ services, click on the option of EC2 as shown in Figure C1.

**
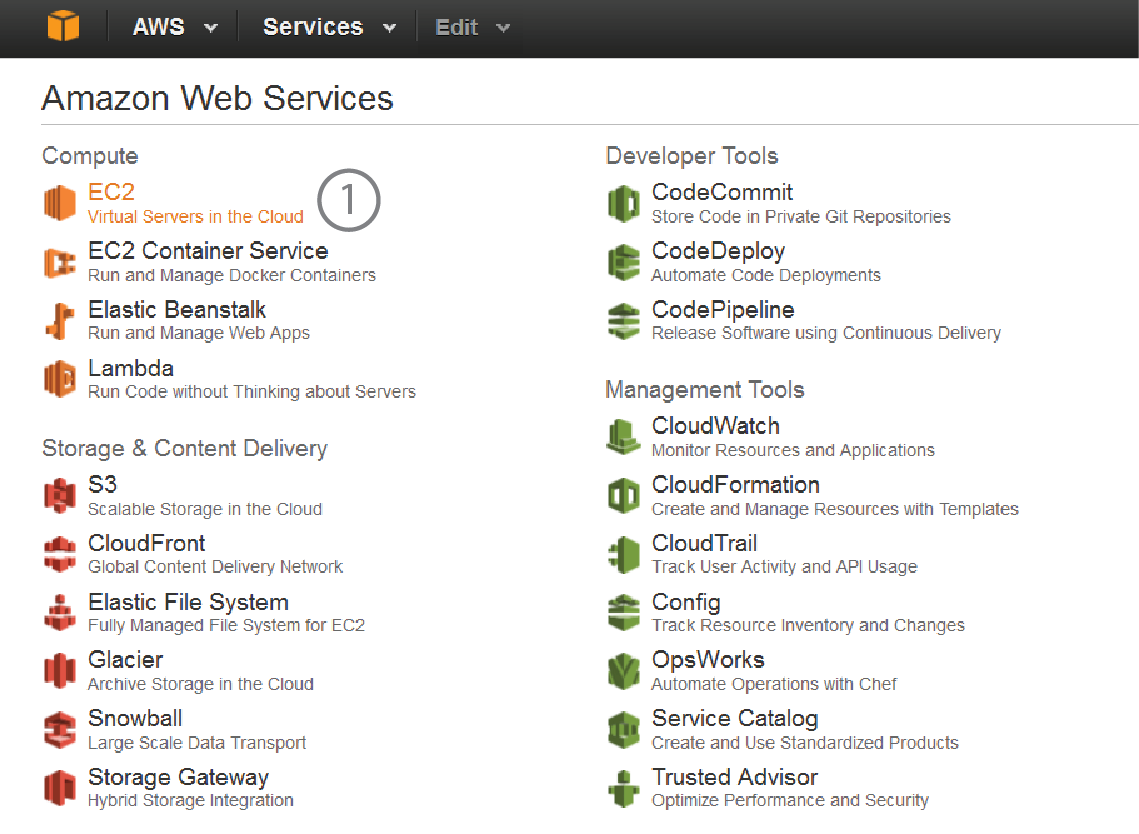
**

**Figure C1: Amazon Web Service page.** This page lists all of the available Amazon Web Services such as ‘Compute’, ‘Storage & Content Delivery’ and ‘Developers Tools’. Click the EC2 (Virtual Servers in the Cloud) (1) to start the web service.

1. Launch Amazon EC2 Instances. To create a virtual server known as an Amazon EC2 instance, click ‘Launch Instance’ from the EC2 dashboard as shown in Figure C2.


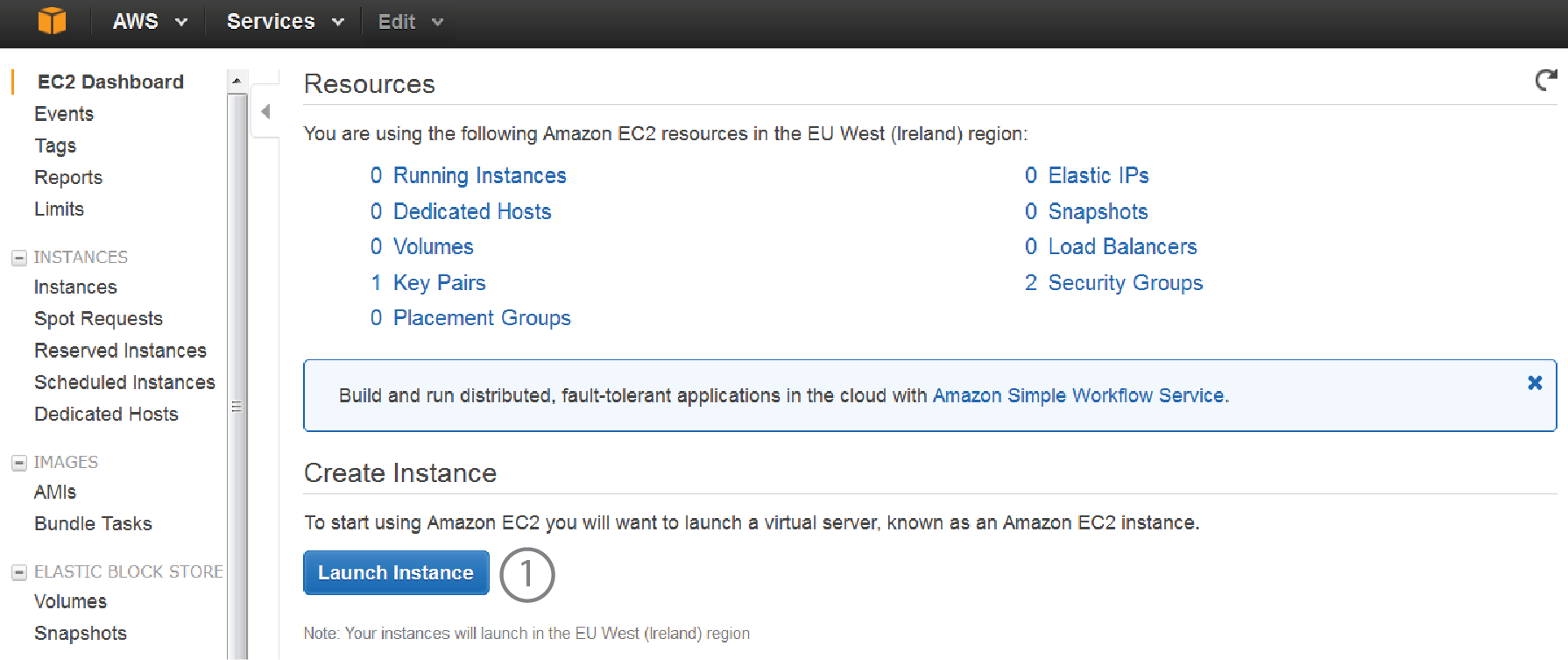


**Figure C2: EC2 Dashboard pages.** This page lists all of the Amazon EC2 resources and a button to create an instance. Click ‘Launch Instance’ (1) to create a virtual server (instance).

1. Launch Amazon Machine Image (AMI). To create a network layout, search ‘NGS Graph Generator’ in the search bar on the Community AMIs, and it will show the application of NGS Graph Generator. Select the application to continue as shown in the Figure C3.


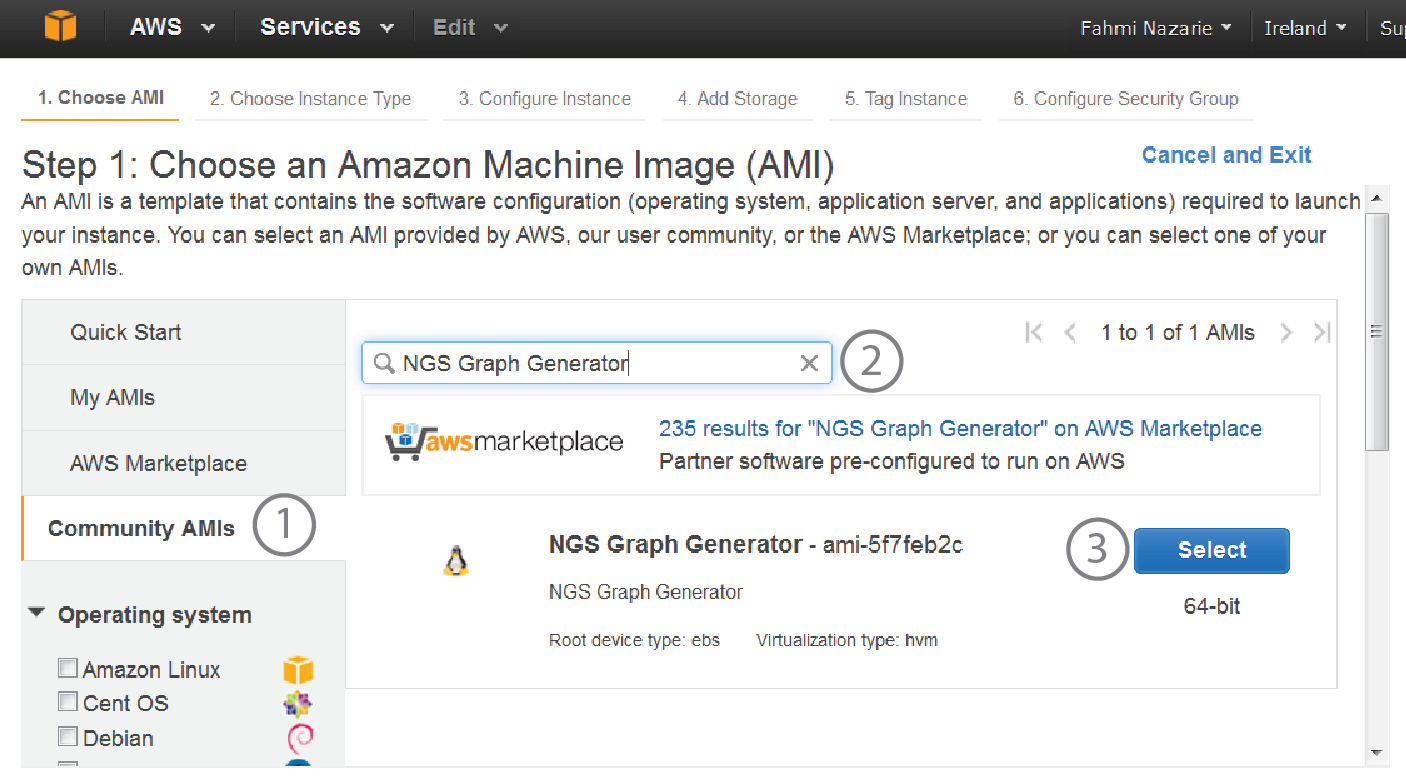


**Figure C3: Choose an Amazon Machines Image (AMI) page.** This page lists all of the available operating system, application server and applications. Under the ‘Community AMIs’ (1) tab on the left, type ‘NGS Graph Generator’ on the search bar (2). The result of the application searched shown and select (3) to continue.

1. Add AMIs into an instance. To add an instance of NGS Graph Generator to your AWS account, you will need to accept the default value of t2.micro, which qualifies for the free tier. (Note: If you select a different instance, you might incur AWS charges.) (Figure C4). The selected value needs to be reviewed before launching the instance (2).


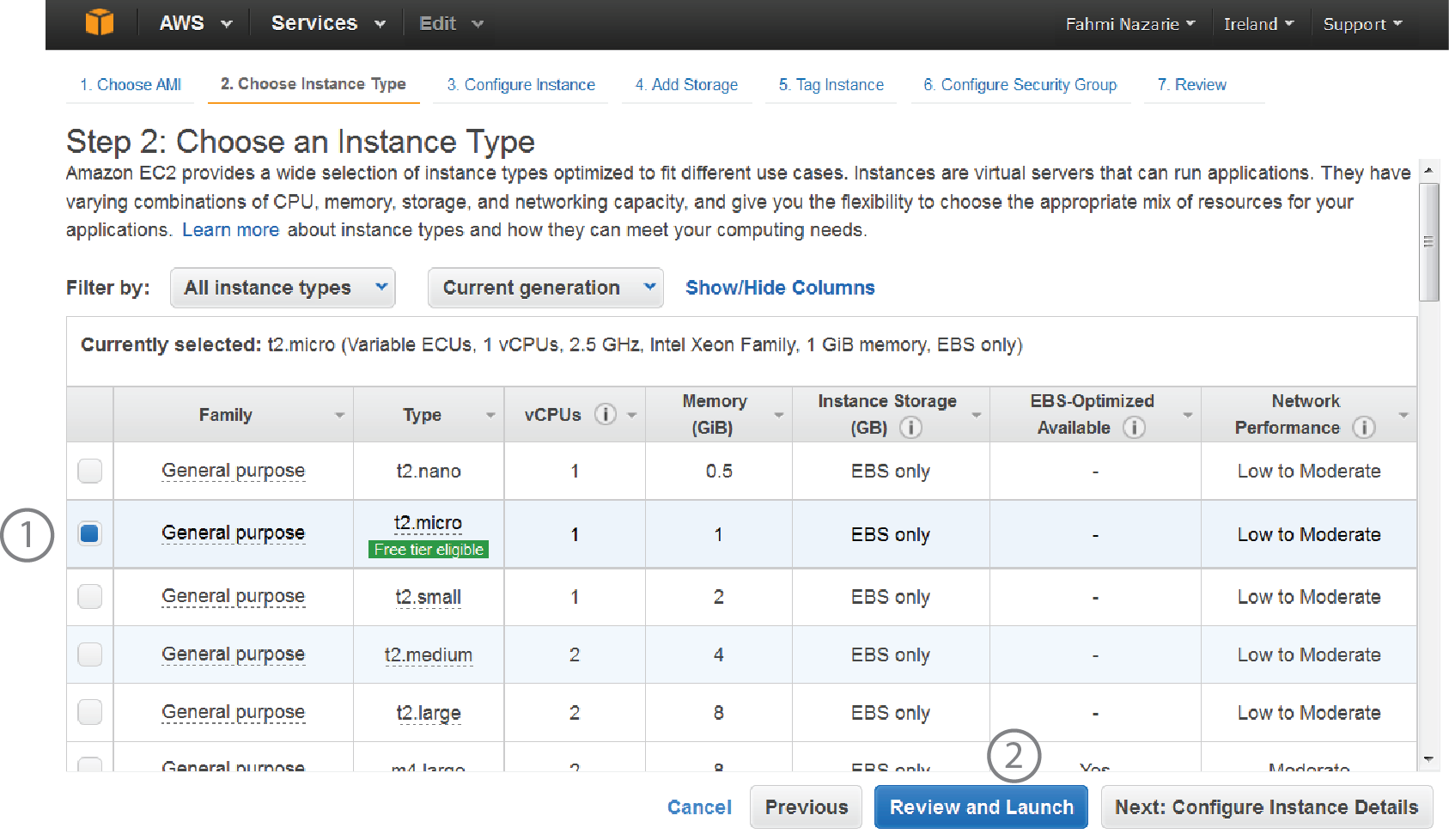


**Figure C4: Choose an Instance Type page.** This page lists all of the available instances type varying combinations of CPU, memory, storage and capacity. In order to use this pipeline, it needs a small memory and low storage, thus a user can select for t2.micro (free tier eligible) (1), then click ‘Review and Launch’ button (2).

After deployment completes, an instance of NGS Graph Generator should appear in your list of software subscriptions (Figure C5). *Note*: You may need to refresh the page to see the NGS Graph Generator instance.


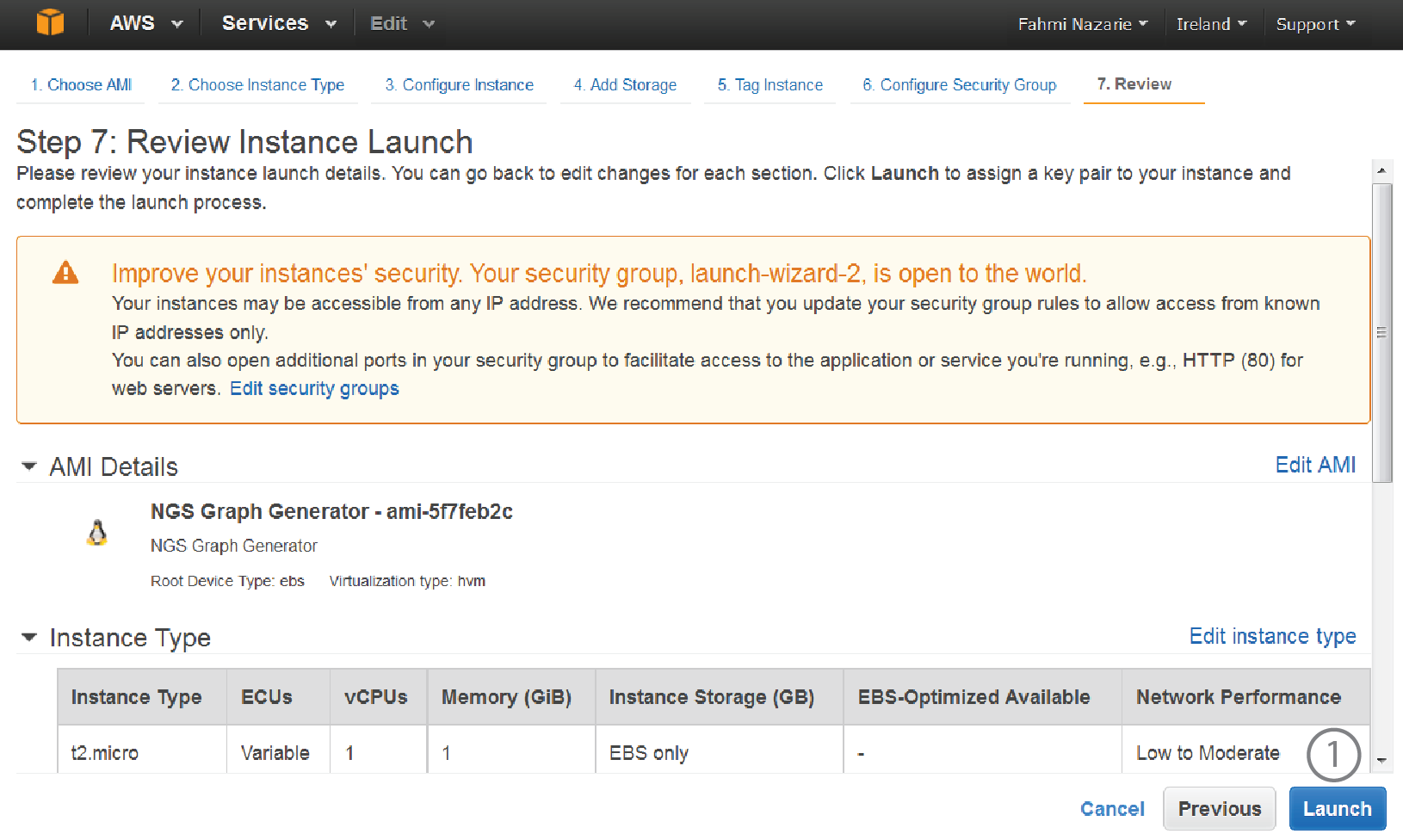


**Figure C5: Review Instance Launch page.** This page shows the selected instance and verifies it before launching then click ‘Launch’ button (1) to start the instance.

1. **Start.** To start NGS Graph Generator:
2. Copy the Public DNS for NGS Graph Generator. In the example, the Public DNA is ec2-54-154-141-216.eu-west-1.compute.amazonaws.com - Figure C6 (1).
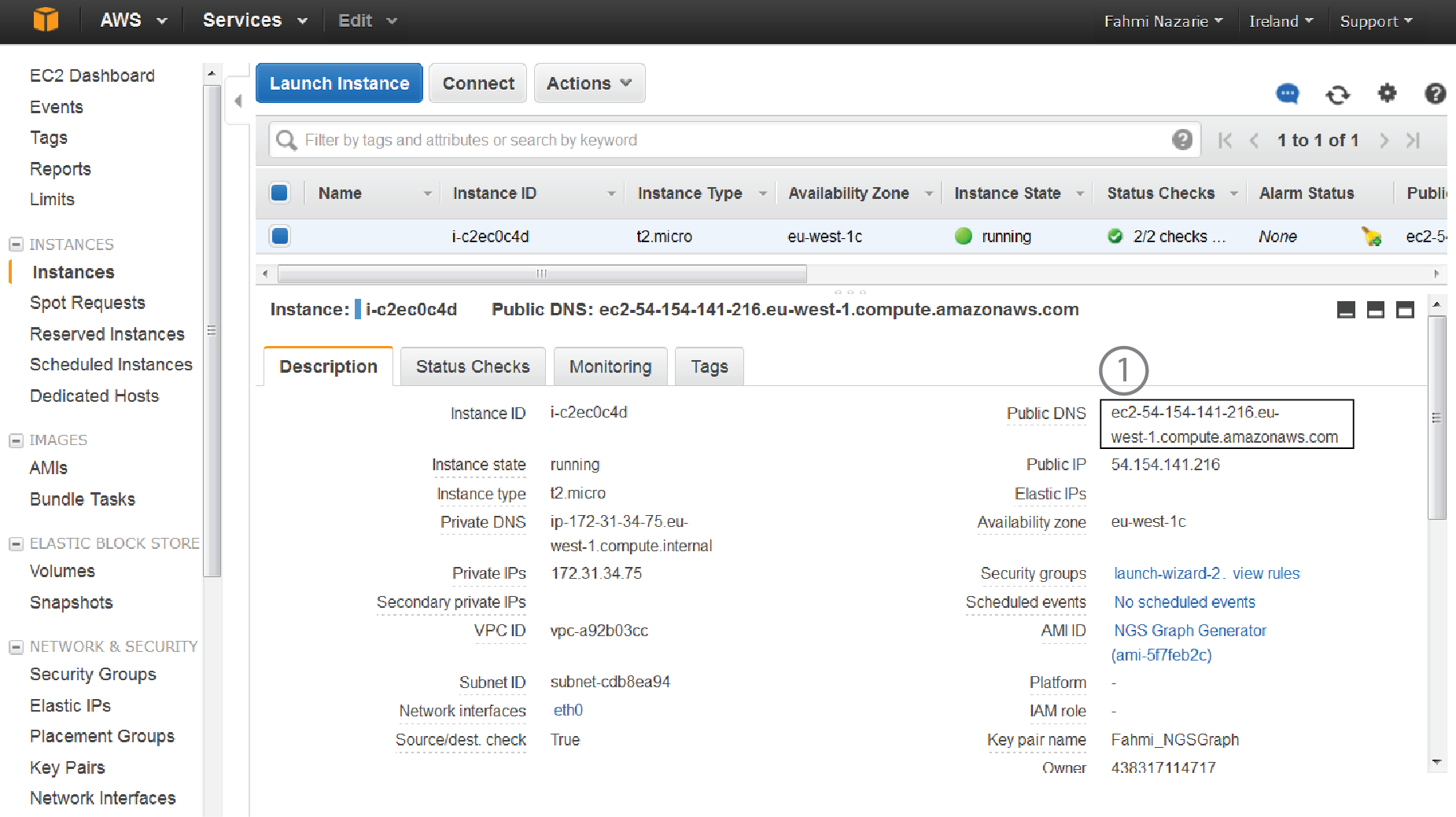


**Figure C6: Instance page.** This page lists all of the information of the launched instance. The Public DNS (1) address is needed for the next step when connecting the instance to Windows.

1. Connect Linux instance from Windows using PuTTY. Download and install PuTTY from <http://chiark.greenend.org.uk/~sgtatham/putty/download.html>
2. Create private key from the PuTTYgen and save it in a local computer.
3. Open PuTTY and the configuration window opens.
4. Paste the Public DNS (in step **a** above) in the ‘Host name or IP address’ blank and save it as ‘AWS’ (Figure C7A(1)).
5. Click ‘Auth’ under that ‘Connection’ and ‘SSH’ (Figure C7B(2)) and the private key previously generated using PuTTYgen is located (Figure C7B(3)).
6. Click ‘Open’ to open the AMIs web server (Figure C7B(4)).


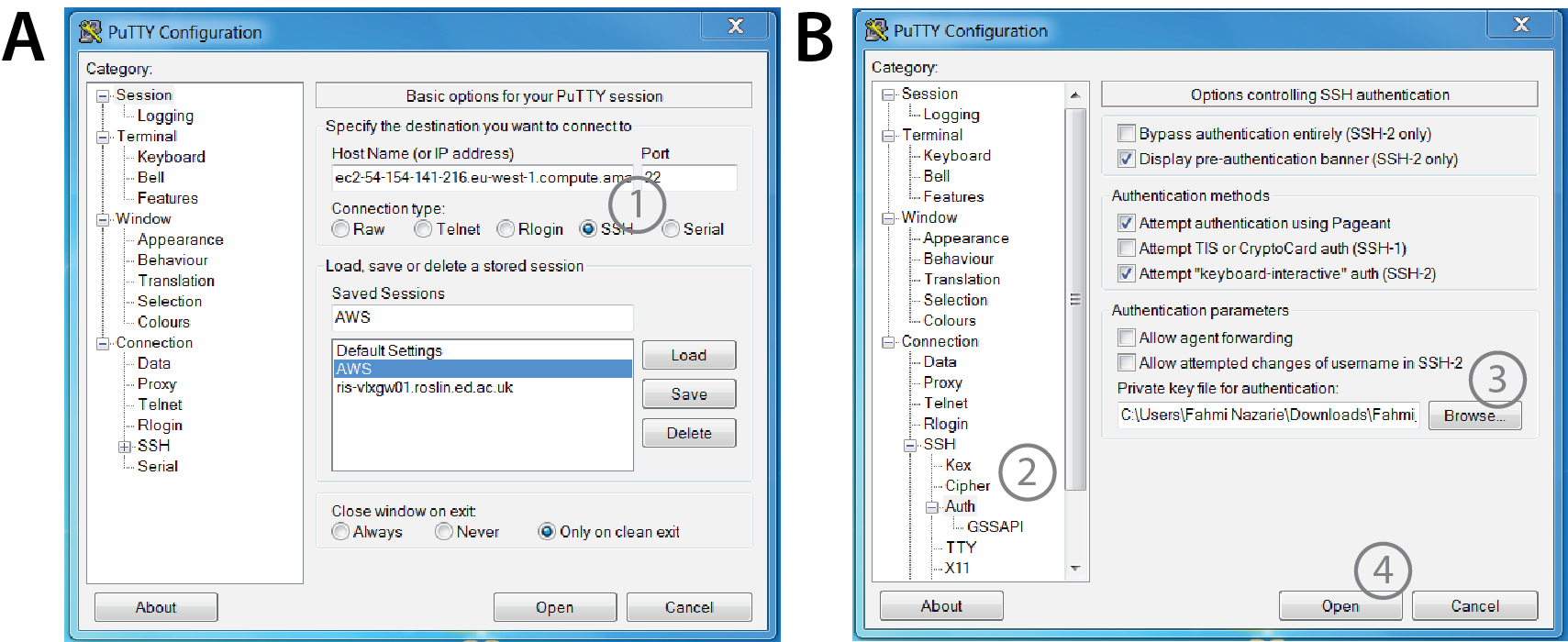


**Figure C7: PuTTY configuration window.** In this windows, only the IP address need to be filled (A1) and save it as AWS. Then, click the ‘Auth’ option (B2) under the ‘Connection’ and ‘SSH’ options. Locate the private key as previously generated using PuTTYgen (B3). Lastly, click ‘Open’ (B4).


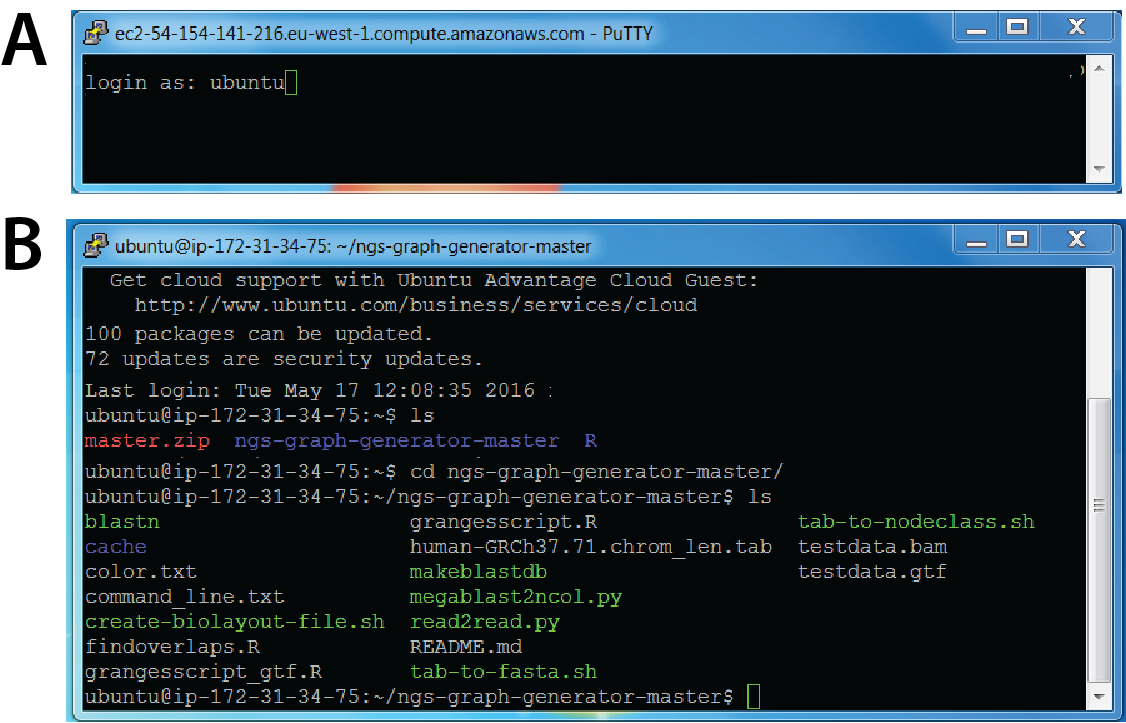


**Figure C8: Windows of connected Amazon EC2 server. (A)** Shows the login window - type ‘ubuntu’ to login. (B) The AMIs of NGS Graph Generator content.

1. **Run the pipeline using our test data.** Once you launch the AMI file in your instances, simply run the pipeline by typing on the server with username “ubuntu” without password (Figure C8). The parameter used here is a default settings (*p=*98*, l=*31). You can change the parameters by increasing their values to increase the stringency of similarity thresholds or vice versa.

$ ./create-biolayout-file.sh –b testdata.bam –t human-GRCh37.71.chrom_len.tab –g testdata.gtf –o output –d LRR1 –p 98 –l 31

$ cd output/

$ ls

1f4f072f18efa9e72fa3376f1d5e17ed

LRR1.fasta

LRR1.nodeclass

LRR1-s98-c31.layout

output.zip

r2r_output

In the ‘output’ folder will be several other files and folders. The network generated from this pipeline will be call ’LRR1-s98-c31.layout’. Visualisation and analysis of the file is explained in the next section.

### Visualizing DNA assembly network files

1. System requirements. The role of the NGS Graph Generator popeline is to generate a layout (.layout) file for visualization on a desktop machine. A layout file for selected gene is a text file containing the weighted edge scores above the selected threshold between all reads mapping to a given locus. It also contains ‘class set’ information which defines the various transcripts to which a node belongs. Layout files are designed to be opened in the software Graphia Professional (Kajeka Ltd., Edinburgh, UK), programs designed for the visualisation and analysis of very large network graphs. Both use relatively high-end 3D visualization technologies and the machine running the program will require a modern graphics card, with preferably at least 4 Gb of RAM and a 64 bit OS, if big networks (>10,000 nodes) are to be explored. The better your machine, the bigger the networks you will be able to render and the smoother your interaction with them.
2. Installation of network analysis tools. Graphia Professional run on Windows, Mac OS X, and Linux platforms. Java SE 6 or 7 is required and can be downloaded from <http://www.java.com/getjava>. Download the Graphia Professional Installer from <http://kajeka.com/graphia-professional/> and download an the appropriate installer for Windows (.exe) or Mac OS X (.dmg). For Linux platform, use the universal JAR file that may be run without an installer. When the tools run for the first time, they create a preferences file that can be changed and saved at any time from the menu option Tools → Save Preferences. Users can customize many options selecting from the menu bar Tools → General Properties (Shift+P). Further details on the software interface and its customization are available in the kajeka.com/wiki/Main_Page that can be downloaded from the tool’s website.
3. Download the layout file. If the layout file is generated from the Website (Section A) – simply click on the file from the download column on the resuls page. If you have run more than two genes, you can simply click the zip file of the job - (number) such job-xxx.gz downloading it onto your computer. Unzip and open the folder to get your layout file. UNIX (Section B) – if you run the pipeline from the server, transfer your file to your desktop using a FTP client such as WinSCP (<https://winscp.net/eng/download.php>). For Mac OS and Linux platforms (i.e. Ubuntu, Fedora, RedHat, etc.), you simply transfer the file to your desktop. Amazon Cloud (Section C) **–** transfer the output file on your computer desktop.
4. Double-click the layout file, e.g. *LRR1-s98-c31*.layout, and it will automatically open in your computer as shown in the software (Figure D1).


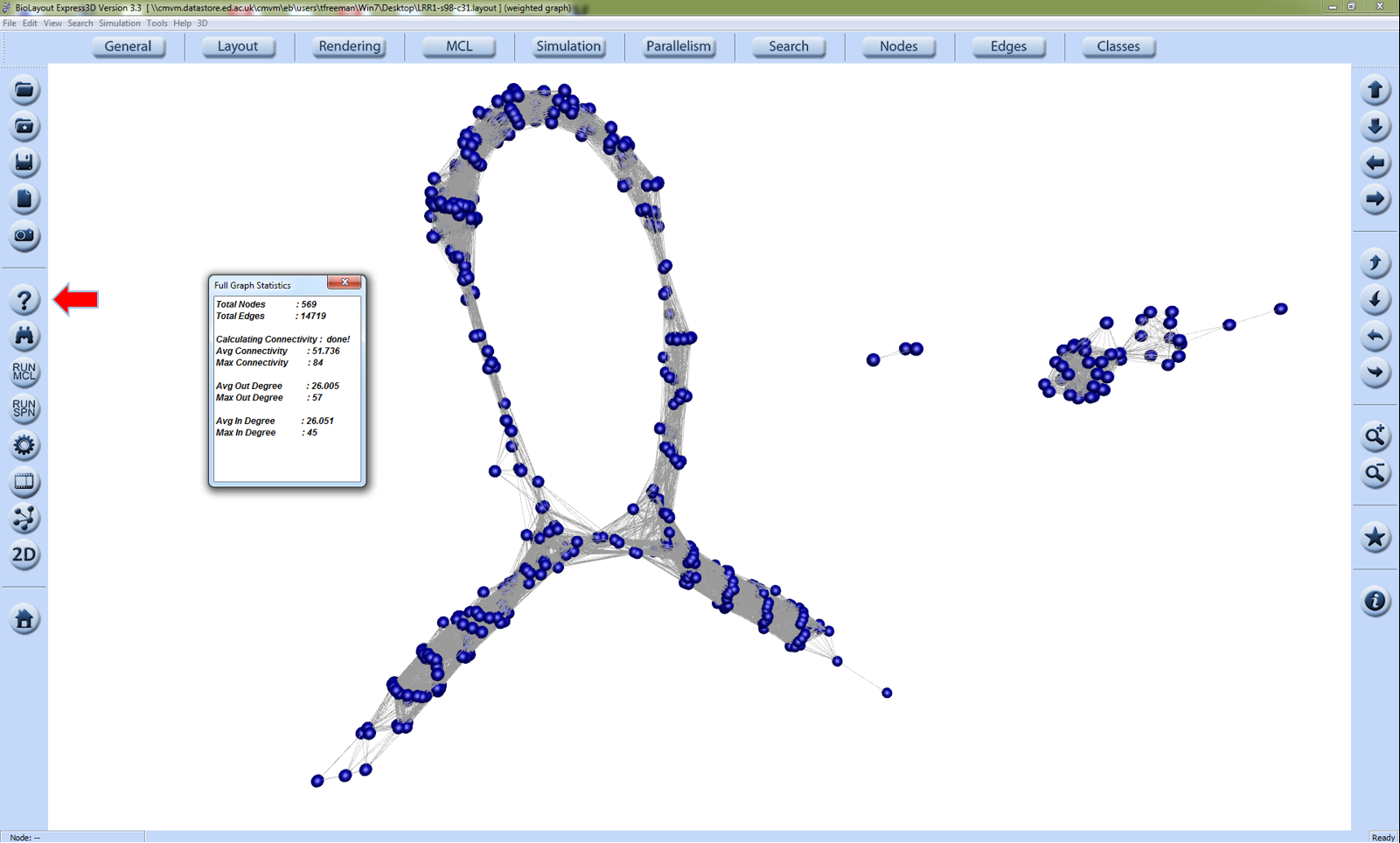


**Figure D1: Layout network transcript of *LRR1* without any exon annotation.** The blue nodes represent DNA sequencing reads whilst each grey line represents a similarity between two nodes above the selected threshold. Graph statistics can be access using the
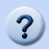
 button on the left (arrow).

1. In the view shown in Figure D1 all nodes are coloured the same, i.e. blue. To understand more about the transcript isoforms represented by this DNA assembly network, go to the *CLASS* tab on top of the screen as shown in Figure D2.


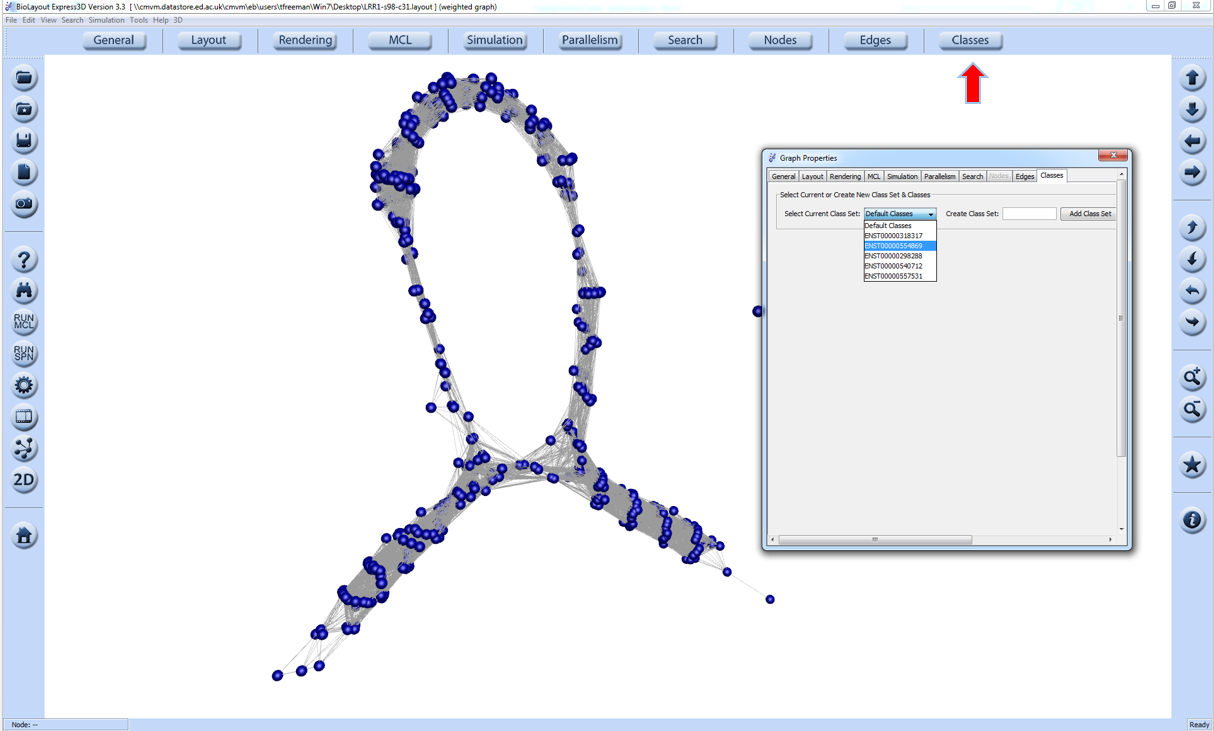


**Figure D2:** The *CLASSES* tab. The Classes tab (arrow) lists the set of transcript models associated with the gene. You can select any of the isoforms to visualise the transcript, but for this protocol *LRR1* isoform ENST00000554869 is selected.

1. Once a transcript model (Class set) has been selected a properties box appears (Figure D3) listing the exons present in the selected transcript and the colour of the nodes that map to them. When a transcript is selected, the colour and class description (exon listing) is shown below. The colour of nodes mapping to a given exon can be changed by clicking on the default colour and changing it using the colour swatch dialogue that appears.


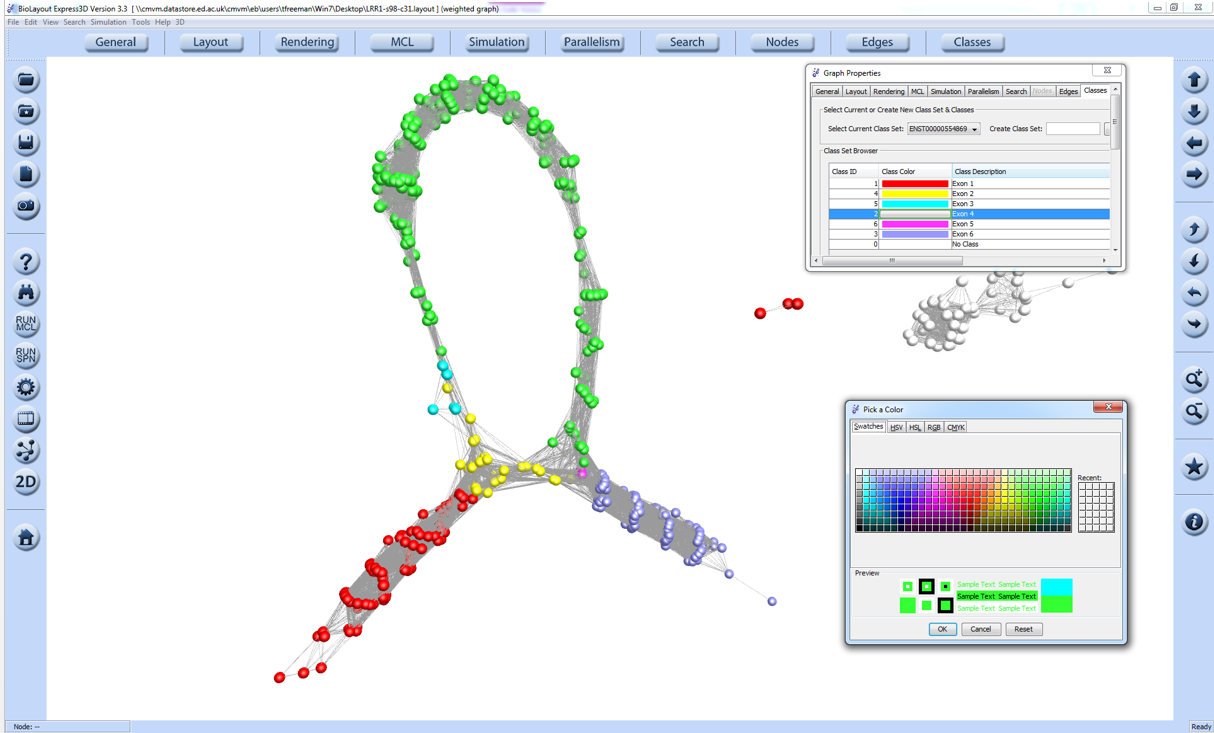


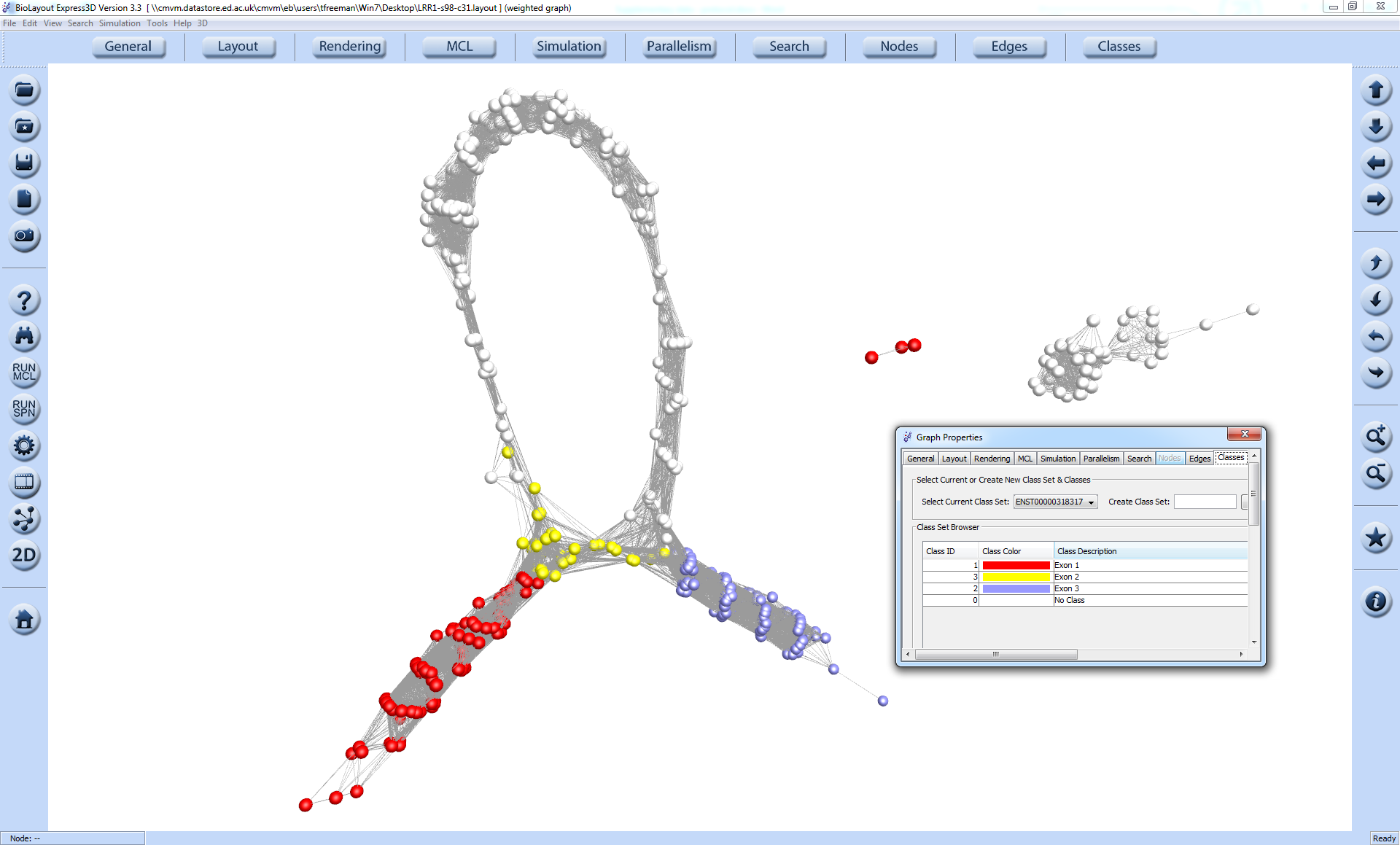


**Figure D3: Class set properties.** *Top:* Having selected a transcript model to visualise from the list, the individual class (exon) colours will be display together with the exon number (description) for this particular transcript model for *LRR1*. If you wish to change the colour of an exon click on a colour and colour swatch will appear. *Below:* visualisation of a different transcript model.

1. A number of options are available for network layout under the Layout tab (Figure D4). By adjusting the settings in this dialogue box the quality of network layout can be altered. The best quality layouts are achieved by using the Eades force model and Very High Quality, Very Low Speeds setting but this comes at a cost of the layout time. Lower quality settings are often sufficient to reveal network structure. For examples of the layout quality and times taken to perform the layout see Figure D5.


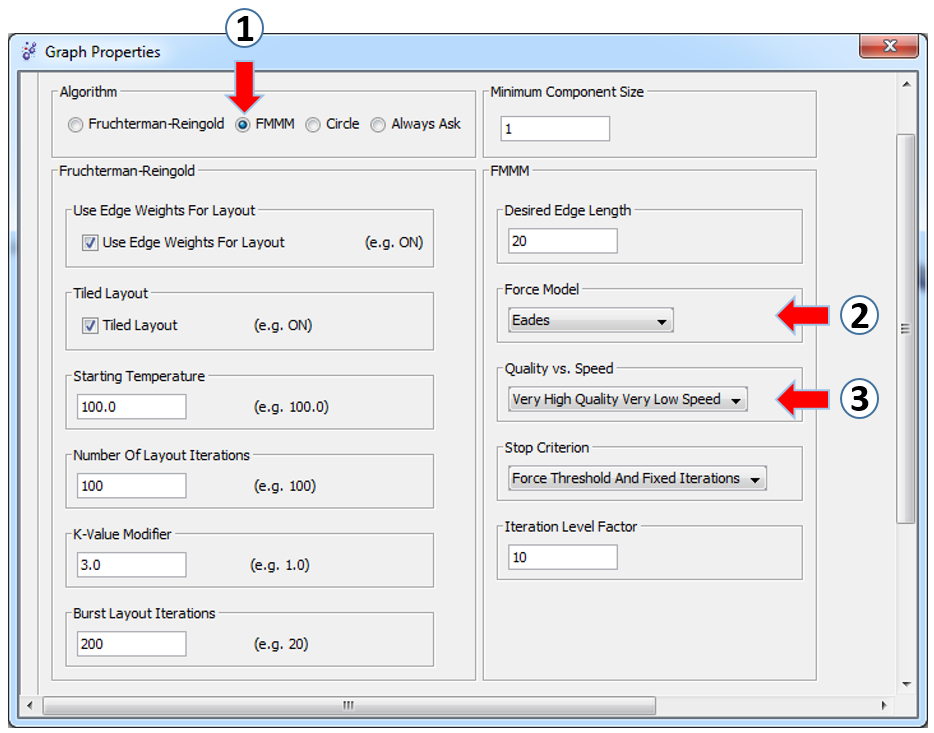


**Figure D4: Layout properties tab.** **(1)** For layout of RNA assembly networks we would recommend that always use the FMMM layout algorithm. **(2)** Selection of force model either Eades or Fruchterman-Reingold. **(3)** Selection of layout quality setting, the higher the quality the longer the layout will take to perform.


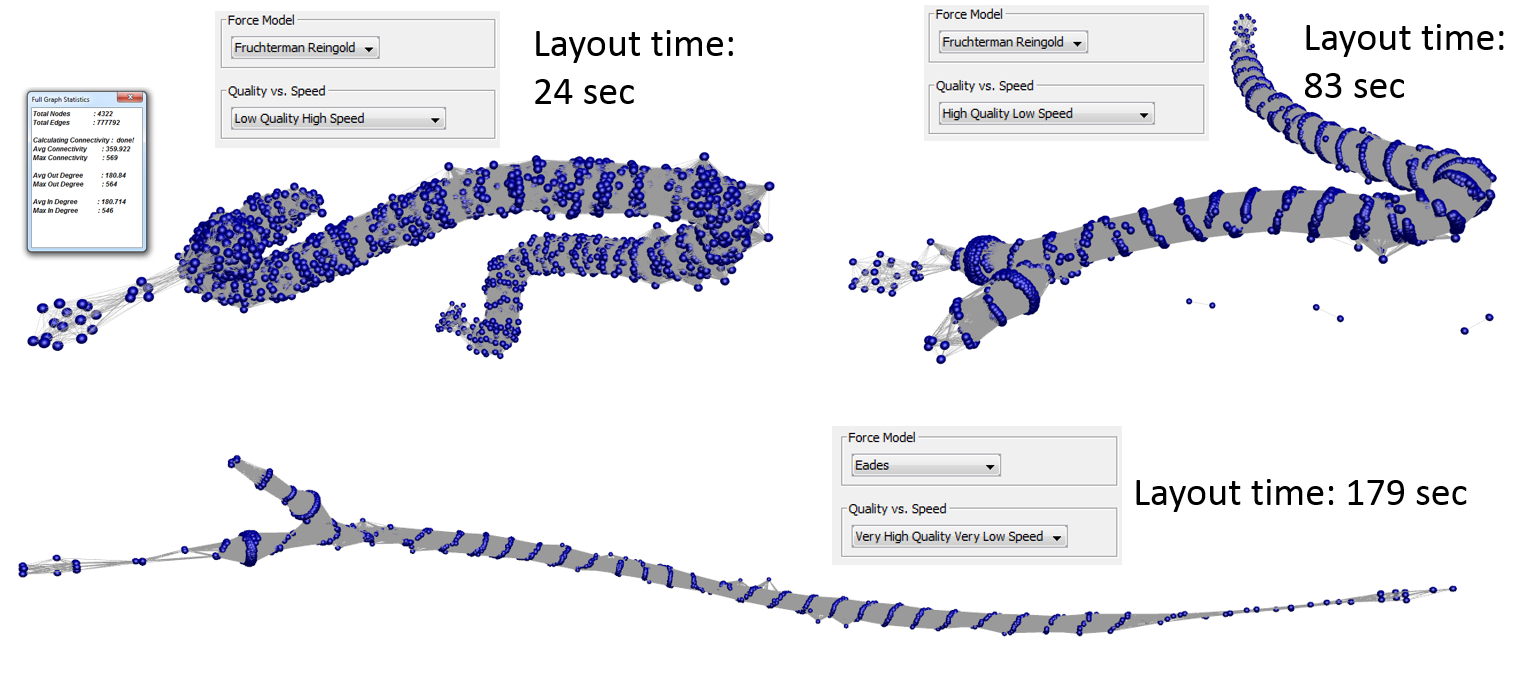


**Figure D5: Examples of graph layout at different quality/force model setting settings.** Gene network *DCN* (decorin) containing 4,322 nodes, 777,792 edges.
