# Supplementary material for: Visualization and analysis of RNA-Seq assembly graphs
Source: Nucleic Acids Res. 2019 Jul 15;47(14):7262–75. doi: 10.1093/nar/gkz599 (PMC6698738; doi:10.1093/nar/gkz599)
Supplement: gkz599_Supplemental_Files [file gkz599_supplemental_files.zip › SUPPLEMENTARY DATA.docx]

SUPPLEMENTARY DATA

Supplementary Figure 1. Optimisation of graph layout. In order to test available alternative layout algorithms, an idealised overlap network was generated by assuming 100 ordered reads (nodes) where each read overlaps the previous read by 95% of its length, i.e. edge weight between adjacent nodes is 0.95. In this paradigm the first read would overlap successive reads by 5% less each time, sharing only 5% similarity to read 20 and no similarity to subsequent reads. A second file of this type was prepared so as to represent two splice variants, where one variant was identical to the first network but a second variant included a 50 node addition where similarities started to branch off after the first 50 nodes, rejoining the graph at position 51. (A) Layout of a perfect overlap matrix consisting of 100 ‘reads’ where consecutive reads overlap by 95% and a >5% similarity has been used as minimum threshold for defining edges. In example Ai a modified Fruchterman-Reingold layout was used to layout the graph, in Aii the FMMM algorithm using the Eades force model and a ‘Low Quality, High Speed’ setting and Aiii is the same as Aii but the ‘High Quality, Low Speed setting’ was used. Aiv is an end on view of Aiii illustrating the corkscrew-like structure of the graph. Av is a visualisation of an alternatively spliced transcript generated from simulated data. (B) Following examination of these synthetic ‘transcript’ graphs a series of tests were performed using data mapping to collagen type 1 V alpha 1 (COL5A1), a long (8.5 kb) and highly expressed gene in human fibroblasts encoded by a single transcript species. This was used to evaluate the performance of the layout algorithm on a large RNA assembly graph. Bi shows the graph layout using the Fruchterman-Reingold algorithm as originally implemented within Graphia Professional and Bii the layout of COL5A1 using FMMM algorithm (Eades force model and the High Quality, Low Speed setting).

Supplementary file 1. Protocol for the generation and visualisation of RNA assembly graphs. Description of pipeline and protocols for generating graphs using the NGS Graph Generator website, Unix, Amazon cloud and for analysing the results.

Supplementary file 2. Video showing visualisation and analysis of RNA assembly graphs.
