# Supplementary figures and images for: Visualization and analysis of RNA-Seq assembly graphs
Source: Nucleic Acids Res. 2019 Jul 15;47(14):7262–75. doi: 10.1093/nar/gkz599 (PMC6698738; doi:10.1093/nar/gkz599)

**A**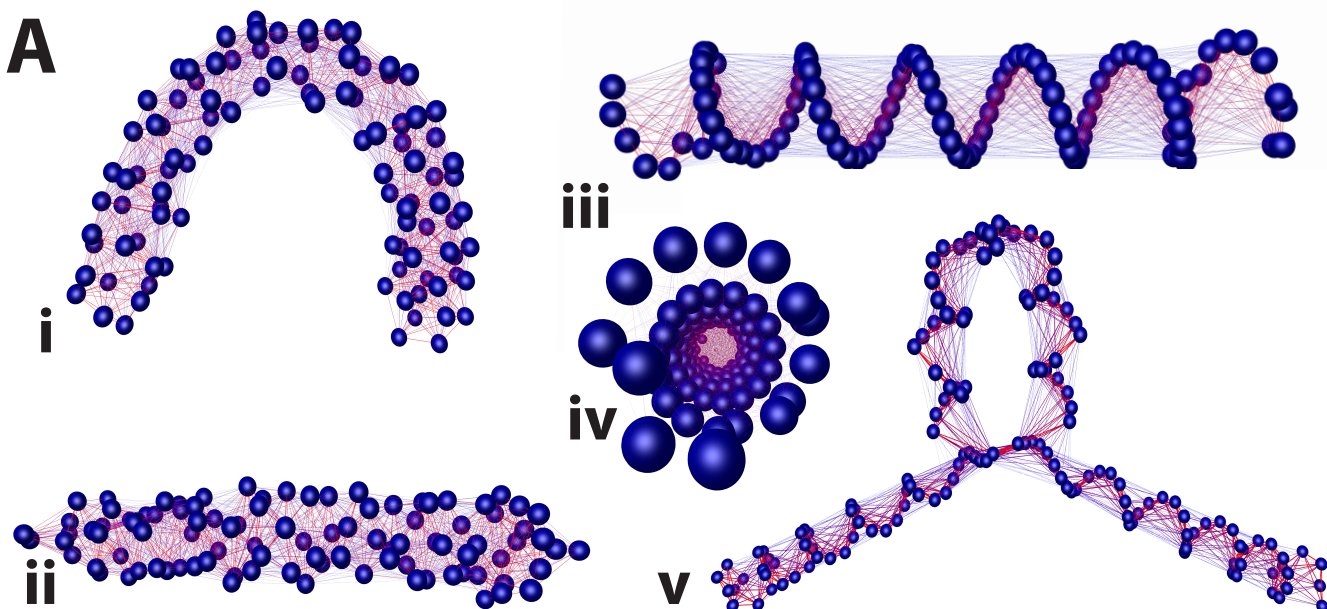**B**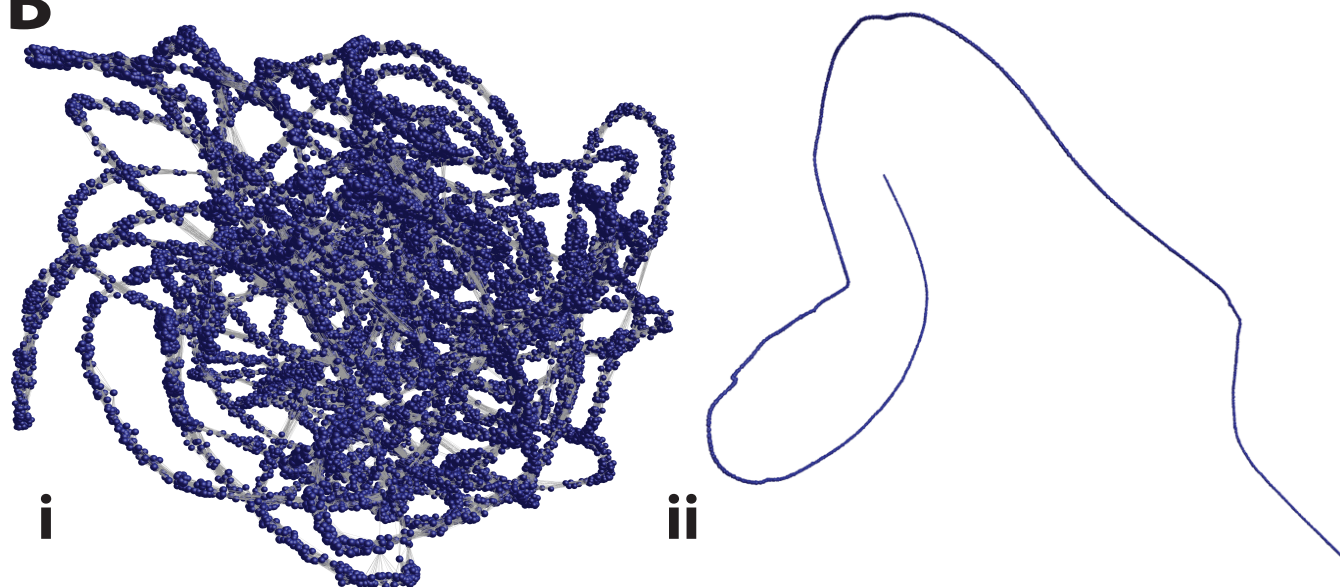

Supplement: gkz599_Supplemental_Files [file gkz599_supplemental_files.zip › Supp. Figure_1.pdf]
